# Autochthonous *Saccharomyces cerevisiae* Starter Cultures Enhance Polyphenols Content, Antioxidant Activity, and Anti-Inflammatory Response of Apulian Red Wines

**DOI:** 10.3390/foods8100453

**Published:** 2019-10-04

**Authors:** Francesco Grieco, Maria Annunziata Carluccio, Giovanna Giovinazzo

**Affiliations:** 1 National Research Council—Institute of Sciences of Food Production (ISPA), via Prov. Lecce-Monteroni, 73100 Lecce, Italy; francesco.grieco@ispa.cnr.it; 2 National Research Council—Institute of Clinic Physiology (IFC), via Prov. Lecce-Monteroni, 73100 Lecce, Italy; maria@ifc.cnr.it

**Keywords:** functional beverage, wine polyphenols, inflammation, winemaking, selected yeast

## Abstract

Several biotic and abiotic factors can influence the amount of polyphenols in grape tissues. During vinification, the temperature, presence of grape seeds and peel, and use of enzymes, can influence the extraction of polyphenols. However, little information is available on the effects of yeast strains used in the polyphenolic composition of wine. With this aim, two selected Saccharomyces cerevisiae strains, ITEM 14093 and ITEM 14077 were used to produce wine from two Italian grape cultivars, Primitivo and Negroamaro. At the end of the alcoholic fermentation, the content of total polyphenols and of particular classes of polyphenols (stilbenes, phenolic acids, flavonols, and flavanols) and the antioxidant activity were evaluated and compared in the obtained wines. We also examined the effects of red wine extracts in a culture model of vascular inflammation. The results obtained comparing wine extracts obtained by utilizing commercial or autochthonous yeast showed that wines obtained with selected yeast significantly inhibited vascular inflammation. The results are positively directed towards the healthy properties of wine drinking.

## 1. Introduction

Grape berries contain a large quantity of dissimilar phenolic compounds in their peel, pulp, and seeds, which are to some extent extracted during winemaking. Red wine polyphenolic extracts are a complex blend of structurally different compounds, flavonoid and non-flavonoid. The diverse polyphenol groups found in wine are significant for determining several technological features of wine [1,2].

Throughout wine production, only part of the flavonoids specifically move from grape to wine, and their final concentration is mainly dependent on the direct contact of the must with the solid parts of the fermenting mixture that include berry peel and seeds [2,3]. Several correlated genetic, agronomic and technological parameters contribute to determining the qualitative and quantitative profile of bioactive compounds in wine [4].

The polyphenol complex of red wines is a source of dietary antioxidants [5]. The red wine polyphenols could operate either as antioxidants or as modulators in the expression of inflammatory human genes [6,7,8,9]. The vascular anti-inflammatory activity of polyphenolic extracts from two characteristic red wines of the Apulia region (South Italy) was defined. Because the oxidative stress can control the expression of inflammatory genes during the atherogenesis process, the intracellular antioxidant potential of the above extracted and pure polyphenols was investigated in inflamed conditions [6]. Studies in vitro and in vivo [10,11] suggested the synergistic capacity of wine polyphenols to address the immune response in the direction of an anti-inflammatory pathway and to stimulate T regulatory cells, thus modulating the atherosclerosis progress and mitigating chronic inflammatory syndromes.

In addition, the inhibitory actions of single polyphenols took place at concentrations higher than those commonly found in wines, thus indicating the presence of a synergist action of different polyphenol mixtures. Furthermore, current investigations demonstrated that pure polyphenols, supplied as dietary supplements, did not produce effects of promoting health similar to those enhanced by the same compounds present in the foods [12].

It should be emphasized that the wine yeast strain exerts not only a prominent role on the organoleptic qualities of wine, transforming aromatic precursors of grape must and producing exogenous aromatic substances, but they also determine the content of polyphenols [13].

To date, three mechanisms of interaction between the polyphenolic component of wines and the yeasts have been recognized [14]. The first mode consists of the adsorption of polyphenols on the yeast cell wall. The quantity of biomass formed during the vinification process is able to entrap a large portion of polyphenol content of fermented must on the cell walls. This property is a strain-specific feature, and this is due to the difference in the composition of the cell wall present in different yeast strains.

The second kind of relation between wine polyphenol and yeast is associated to the microbial β-glucosidase enzymatic activity, since this enzyme is able to break the polyphenols–sugar bond, thus modifying the chemical and antioxidant profile of the obtained wine.

The third possible microbial action on the polyphenolic content of wines concerns the secretion during the vinification process by specific yeast starter strains of polysaccharides able to establish stable complexes with polyphenols. Several studies investigated the features of wines produced by the separated inoculation of diverse *S. cerevisiae* strains, showing that the strains were able to adsorb polyphenolic compounds on their cell walls during the alcoholic fermentation process [13,15]. This investigation employed dissimilar yeast strains to perform vinification tests, and these starters were confirmed to be able to influence specially the trans-resveratrol concentration and antioxidant capacity in the obtained wines.

Similar results have been recently obtained by testing singular yeast strains for Pinot Noir [16], Albariño [17], and Gaglioppo [18] wines, thus confirming that the above starters were capable of making wines provided with a specific polyphenol composition.

Some studies have been conducted to demonstrate the possible effects of the selection criteria for oenological yeast on the quantitative composition of the phenolic compound of wines [19,20]. The obtained evidence indicated that a particular vine, by virtue of its phenolic composition, modulates the parietal adsorption activity of a specific yeast strain.

During previous studies, two populations each consisting of 1000 different isolates of *Saccharomyces cerevisiae* were isolated during the last step of the spontaneous alcoholic fermentation of Primitivo [21] and Negroamaro [22], respectively, and then subjected to an oenological selection procedure [23]. Two different strains, namely ITEM 14093 and ITEM 14077, were identified as a “microarea-specific” starter culture for Primitivo and Negroamaro wine production, respectively.

The obtained wines were compared for the antioxidant activity of total polyphenols and composition of specific classes of phenolic compounds. We studied the effects on certain polyphenol classes abundant in the above red wines, such as flavonoids, namely flavonols, as well as non-flavonoids, such as phenolic acids and stilbenes. Similar analyses were carried out in parallel on wines produced form the same grape must using commercial yeast strains. This paper reports an attempt to verify the improved health promoting features of produces wines, by comparing polyphenols composition and anti-inflammatory activity of wines obtained by utilizing commercial and autochthonous yeast strains. The aim of this research was to study in depth the role of native yeast strains in improving the polyphenol content, antioxidant activity, and anti-inflammatory property of wines produced in the same geographical area of production from which they were isolated. To our knowledge, this study is the first investigation about the influence of autochthonous yeast culture associated with the two most important wine-producing areas of Apulia on the polyphenol composition and health characteristics of Primitivo and Negroamaro wines.

## 2. Materials and Methods

### 2.1. Reagents

Trans-piceid and trans-resveratrol were purchased by ICN Biomedicals (South Chillicothe Road, Aurora, OH, USA), whereas kaempferol, myricetin, catechin, epicatechin, and oenin were obtained from Extrasynthese (Genay, France). Caffeic acid, quercetin, caftaric acid, p-coumaric acid, and other compounds when not otherwise specified were supplied by Sigma-Aldrich (St. Louis, MO, USA).

### 2.2. Wine Production

The yeast biomasses were produced employing the fermenter Biostat C (Sartorius, Germany) according to Tristezza et al. [23]. The starting inoculum (1.5 × 10^6^ CFU/mL) was added to 300 L of Primitivo or Negroamaro must and incubated for 6 h. After that, the above mixture was mixed with 120,000 L of the respective must in a 150,000 L stainless steel vat. The vinifications were performed at 25 °C and their dynamics were controlled every day by assessing the residual sugar concentration. When the alcoholic fermentation process was completed (0°Babo), wine samples were saved for additional analyses. Wines were physicochemically characterized by Fourier transform infrared spectroscopy (FTIR), using the WineScan Flex (FOSS Analytical, Hillerød, Denmark). The industrial tests were carried out on Primitivo must using the strain ITEM14093 in three wineries positioned in Cassano delle Murge (P1A, etc) and Locorotondo (P3A), both being located in the “Gioia del Colle” DOC area (Apulia Region; Southern Italy). The large-scale vinifications of Negramaro must were conducted by employing the yeast strain ITEM 14077 in three industrial cellars situated in the Salento Area (South Apulia) and denoted as N1A, etc. When the alcoholic fermentation ended, the yeast populations were sampled and identified by inter-delta typing at the strain level, according to Tristezza and coworkers. [24]. In particular, obtained data were reported in a binary format with “1” for the existence of a band/peak and “0” for its lack. A matrix of similarities between each pair of individuals was created using the Dice’s similarity index, and it was used to build a UPGMA dendrogram, using the SAHN-clustering and TREE programs (NTSYS-2.1; Applied Biostatistics, Setauket, New York, USA).

Each of the six vinifications was carried out in parallel with a similar vinification, performed by inoculating the same must with the commercial starter normally employed in each of the six industrial wineries. The three Primitivo wines produced by a commercial yeast starter were denoted as P1C, P2C, and P3C, whereas the Negroamaro wines similarly produced were called N1C, N2C, and N3C.

The most important physicochemical parameters of the produced wines were evaluated by FT-IR to determine the main chemical parameters (Table 1). These obtained data were similar to those achieved by the analysis of the wines obtained with the commercial starters indicating that the ITEM14093 and ITEM 14077 strains were able to correctly carry out the alcoholic fermentation processes.

### 2.3. Red Wine Polyphenol Extraction and HPLC Analysis

The wines were analyzed shortly after bottling (7 days) as per Calabriso et al. [9]. Wine samples (10 mL) were extracted three times with methanol 99% and methyl tert-butyl ether (MBE) in a ratio of 2:1:1 (wine:methanol:MBE, *v*/*v*/*v*). Supernatants were collected in fresh tubes and evaporated at 35 °C to dryness.

Wine extracts were analyzed quali-quantitatively using a 1100 Series HPLC system (Agilent) equipped with a Luna 5 µm C18 (2) 100 Å column (250 × 4.6 mm) (Phenomenex, Torrance, CA, USA). The mobile phases were acetonitrile (A) and 1% (*v/v*) H_3_PO_4_ in water (B), with a linear gradient from 20% to 60% acetonitrile in 40 min. The flow rate was 1.0 mL/min, and the column temperature was maintained at 25 °C [25]. The metabolite concentrations were obtained by deduction from the calibration curves and expressed in mg/L. The wavelengths used for the quantification of phenol compounds were 290, 306, 320, 370, and 520 nm. Recovery was determined for the overall assay by adding known amounts of different metabolites to the original concentration of the analyzed samples and the obtained values were between 85% and 93%.

The identification of individual phenol was performed based on their retention times and spectroscopic spectrum. Stock solutions were prepared by dissolving weighted amounts of each standard compound in 80% (*v/v*) methanol-water mixture. These solutions were individually injected into the HPLC column and eluted with the above gradient elution method to determining their chromatographic retention times and collecting UV spectra. The stock solutions were diluted with the 80% (*v/v*) methanol–water mixture to obtaining working solutions of each compound covering ten points of the regression curve. Quantification of individual compounds was performed using a ten-point regression curve of the UV absorption data collected at the wavelength of maximum absorbance of each analyte. (Concentration ranges: chlorogenic acid, caffeic acid, caftaric acid, ferulic acid, cinnamic acid, gallic acid, kaempferol, quercetin-glucoside, catechin, and epicatechin, 500–0.48 mg/L; trans-piceid, coumaric acid, kaempferol glucoside, myricetin, 250–0.24 mg/L; trans-resveratrol, quercetin, 125–0.122 mg/L; oenin 1–0.0095 g/L).

### 2.4. Total Polyphenols Content

The total amount of polyphenols was measured by the optimized Folin–Ciocalteu method [26]. The total phenolic content in wine extracts was determined by measuring the absorbance at 765 nm according to the Folin–Ciocalteu colorimetric method. Results were expressed as milligram gallic acid equivalents per liter (mg GAEs/L).

### 2.5. TEAC Antioxidant Capacity Determination

The Trolox equivalent antioxidant capacity (TEAC) assay is based on the scavenging ability of antioxidants to quench the radical cationic activity of 2,2′-azinobis (3-ethylbenzoithiazolone 6-sulphonate) (ABTS•+). The assay was performed as previously described [25] with some modifications. To generate the ABTS•+ radical cation, ABTS was dissolved in water (7 mM) and incubated with 2.45 mM potassium persulfate (final concentration) in the dark at room temperature for 12–16 h before use. For the calibration curve, the ABTS•+ solution was diluted with water to an absorbance value of 0.70 (± 0.02) at 734 nm and mixed with 20 μL of Trolox standard solutions (from 0 to 25 μM). The assay was performed with extracts from wine, and absorbance was determined at 734 nm. Values were expressed as μmol Trolox equivalents (TE)/L.

### 2.6. Cell Culture and Treatment

Human micro-vascular endothelial cell line (HMEC-1) was obtained from Dr. Thomas J. Lawley and was cultured as described [27]. Confluent endothelial cells were shifted to the medium (MCDB-131) supplemented with 3% fetal bovine serum (FBS). Subsequently, cells were pre-treated for 1 h with polyphenolic extracts obtained from a blend of wines of Negroamaro, NC (N1C, N2C, N3C blend), or Primitivo, PC (P1C, P2C, P3C blend), produced with industrial yeast or obtained by autochthonous yeast from Negroamaro, NA (N1A, N2A, N3A blend), or Primitivo, PA (P1A, P2A, P3A blend), respectively. Then, endothelial monolayers were stimulated with the bacterial endotoxin lipopolysaccharide (LPS) (0.5 μg/mL) for an additional 16 h, after which cellular toxicity, inflammatory markers, and an adhesion assay were evaluated.

### 2.7. Cytotoxicity Assays

Cell viability was assessed by using the dye MTT (3-4, 5-dimethylthiazolyl-2)-2, 5-diphenyltetrazolium bromide) as previously described [28]. The assay is based on the ability of living cells to convert MTT into an insoluble purple colored formazan, the amount of which is proportional to the number of living cells. Cells seeded in 96-well tissue culture plates at a density of 10.000 cells/well were exposed to wine blend polyphenolic extracts (NC, PC, NA, PA) for 1 h and then treated with LPS for a further 16 h. After treatments, endothelial cells were incubated with MTT (5 mg/mL) at 37 °C for 4 h. After discarding the medium, the formazan dye was extracted with DMSO/ isopropanol and absorbance was read at 570 nm with a reference at 690 nm. The absorbance values of control cells were set as 100% viability and MTT conversion of treated cells was expressed as a percentage relative to the control cells.

### 2.8. Endothelium-Monocyte Adhesion Assay

The human monocytic cell line U937 was purchased from the American Type Culture Collection (Rockville, MD, USA) and grown in RPMI medium 1640 (Gibco BRL, Gaithersburg, MD, USA) containing 10% FBS. HMEC-1 were grown to confluence in 6-well tissue culture plates until confluence and then pre-incubated for 1 h with wine blend polyphenolic extracts (NC, PC, NA, PA) before stimulation with LPS (0.5 μg/mL) for an additional 16 h.

U937 cells were labeled with 1 µmol/L calcein-AM (Molecular probe) for 30 min in RPMI medium 1640 (Gibco BRL, Gaithersburg, MD, USA) containing 3% FBS. In a co-culture system, labeled U937 were seeded at 5 × 10^5^ cell density onto an HMEC-1 monolayer and incubated under rotating conditions (63 rpm) at 21 °C as described [8]. After washing, the fluorescence intensity of adherent U937 in each well was measured in a microplate reader with an excitation/emission wavelength of 485/530 nm.

### 2.9. Detection of Endothelial Cell Surface Expression of VCAM-1

Endothelial cells were grown on 96-well tissue culture plates until confluence and then pre-incubated for 1 h with wine blend polyphenolic extracts (NC, PC, NA, PA) before treatment with LPS for a further 16 h. Endothelial surface expression of vascular cell adhesion molecule (VCAM)-1 was assayed by employing a cell surface enzyme immunoassay (EIA) using primary mouse anti-human monoclonal antibodies against VCAM-1 (Millipore).

### 2.10. Statistical Analysis

Three samples were utilized for each group and all tests were done in triplicate. The results were shown as mean values and standard deviations (SD). Dissimilarities between two groups were calculated by unpaired Student’s *t*-test. Multiple comparisons were carried out by one-way analysis of variance (ANOVA) and by Tukey’s test with *p* = 0.05. Dissimilarities between means from at least three independent tests (*p* < 0.05) were judged statistically significant.

## 3. Results

### 3.1. Industrial Vinification and Comparison of Chemical Parameters of Negroamaro and Primitivo Wines Obtained with Commercial or Autochthonous Yeast

In Primitivo wines, ethanol concentrations (g/100 mL) varied from 15.87 (P2) to 13.39 (P1), whereas in Negroamaro wines the ethanol amount varied from 13.72 (N2) to 13.10 (N1) (Table 1). The sugar content determined for all the obtained wines was always found below 2.5 g/L, a concentration corresponding to a completed fermentation [21]. The total acidity determined in the obtained wines showed content ranging from 5.93 g/L to 5.22 g/L, whereas the levels of volatile acidity (VA) varied from 0.36 g/L to 0.43 g/L. In all wine, VA was found minor than the 0.6 g/L value. A concentration above this significant value is undesirable because it increasingly gives an acetic taste to the wine [29]. The two yeast starters produced a satisfactory quantity of glycerol, ranging from 8.89 to 10.01 g/L in Primitivo wines and from 8.67 to 8.41 g/L, in Negroamaro wines.

**Figure 1:**
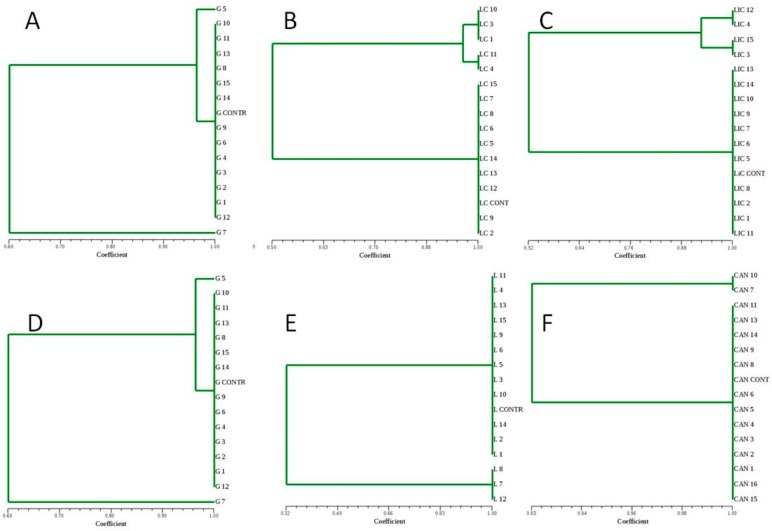
UPGMA dendrograms generated by cluster analysis of inter-δ region patterns obtained from the *Saccharomyces cerevisiae* strains isolated during the later stages of six different large-scale vinifications obtained from: Primitivo grape must PA1 (**A**), PA2 (**B**), and PA3(**C**) inoculated with the 14093 strain; Negroamaro grape must NA1 (**D**), NA2 (**E**), and NA3(**F**) inoculated with the 14077 strain. The genomic DNA extracted from a pure culture of the 14093 and 14077 strains have been used as a control (CONTR).

The ITEM 14093 and ITEM 14077 strains were able to dominate the six different vinifications, as indicated by the evaluation of polymorphism of the inter-δ region (Figure 1).

### 3.2. Comparison of Polyphenols in Negroamaro and Primitivo Wine Extracts Obtained With Commercial or Autochthonous Yeast

Stilbenes content increases in grape from veraison to ripening, with important variations among *V. vinifera* cultivars [30]. These substances, and in particular resveratrol, are compounds related with the valuable effects of consuming wine. The most important stilbenes present in grape are resveratrol-3-O-β-D-glucopyranoside (piceid), cis- and *trans*-resveratrol (3,5,4′-trihydroxystilbene), resveratrol dimers (viniferins), and piceatannol (3,4,3′,5′-tetrahydroxy-trans-stilbene) [14]. The stilbenes glycosylation is important for their translocation, antifungal activity, and preservation against oxidative degradation. In Table 2 and Table 3 the results related to the analysis of stilbene levels in Primitivo and Negroamaro wines obtained by the same grape musts by using commercial starters (PC and NC) and selected autochthonous starters (PA and NA) are shown. 

Resveratrol was present in all the analyzed wines, both in the free form (*trans*-resveratrol) and in the glycosylated form (trans-piceid). The total concentrations of stilbenes were significantly increased either in Primitivo or Negroamaro wine obtained by autochthonous yeast fermentation compared to commercial wines (Table 2 and Table 3). The increase of total stilbenes, in P1A, P2A, and P3A was not significantly different (4.59-, 5.55-, 4.97-fold, respectively) but interestingly higher than commercial wines. Among the Negroamaro wine, the N1A sample obtained with indigenous yeasts had a 2.75-fold total stilbenes increase, which was lower than the other two wines N2A and N3A (3.63- and 3.28-fold increase, respectively).

Phenolic acids are the object of rising attention in health applications due to their antioxidants and anti-inflammatory properties [9,31]. These substances are antioxidant molecules operating through radical scavenging activity thanks to their electron- or hydrogen-donating property and their capability to stabilize/delocalize the resultant phenoxyl radical inside their structure. 

In the chromatographic profile of the wine extracts the caffeic, the caftaric, and the coumaric acids were quantifiable (Table 2 and Table 3). The three metabolites had a similar relative distribution in Primitivo wines, which became more evident when the wines obtained by commercial (PC) or autochthonous yeast (PA) were compared, showing a slight increase in the P1A (2.19-fold compared to 1.95- and 1.7-fold in P2A and P3A, respectively). Among the wines obtained with the autochthonous starter, those produced with Negroamaro grapes contained total amounts of soluble acids significantly higher in comparison with NC wines (N1A: 2.48-fold, N2A: 3.41-fold, N3A: 5.47-fold).

Grape flavanols are present in wine as either monomers (catechin, epicatechin, gallocatechin, epigallocatechin, and epicatechin 3-gallate) or as both oligomers and polymers, also called tannins and proanthocyanidins. Flavanols have proved to be potent antioxidants in different in vitro models, and in some in vivo studies [2,5]. However, their potential beneficial effect on cardiovascular health is not merely due to this property but includes the different mechanism implicated on cardiovascular conditions. Given that wine has relatively low levels of these compounds, with some varietal differences, it is unlikely that manipulation of levels will reach anywhere near those used in toxicology studies. Wines may be enhanced in catechin through viticulture or vinification processes. Catechin and epicatechin are the flavanols identified in our analyzed wines (Table 2 and Table 3). By comparing results, no changes were found in the flavanols content in either Primitivo or Negroamaro wines obtained by commercial or autochthonous yeast.

Flavonols are important in wine co-pigmentation together with anthocyanins, and an important chemo-taxonomical parameter [3]. Flavonols are considered bioactive compounds of possible importance for human health and nutrition. Flavonols accumulated in grape skins and stems as several different glycosides since they protect against damage from ultraviolet (UV) light [32]. Moreover, quercetin glycosides and anthocyanins may be hydrolyzed in wine to form aglycons. Flavonols can interact with anthocyanins, enhancing their red color in a process known as co-pigmentation.

The consumption of flavonol-rich diets has a preventive function against cardiovascular diseases certain cancers, and age-related diseases in the animal and human epidemiological studies [5,33]. This preventive activity results from both the action of flavonols as controllers of antioxidant defenses and their direct inhibitory effects on cellular proliferation. Both the absolute quantity and the distribution of the flavonols changes in an important way during vinification; only a part of the flavonols present in the grapes at harvest is found in the corresponding wines. In particular, other authors also have found that an average loss of about 85% of flavonols from grape to wine occurs [3]. Myricetin, quercetin, and kaempferol are the flavonols identified in our analyzed wines (Table 2 and Table 3). For this class of compounds, in wines obtained from autochthonous yeast fermentation, the relative concentrations of the individual flavonols are higher than those determined in commercial wines. The relative distribution of the compounds varied according to the extract analyzed. In all Primitivo wines, the amount of flavonols increased when fermented with autochthonous starters, with the highest percentage of increase in the P1A sample (5.48-fold increase). By comparing results, also in Negroamaro samples an increase in total flavonols of 3.92-fold in N2A and 7.5-fold in N3A was found, whereas in N1A, no changes were found in the flavonols content.

### 3.3. Comparison of Total Polyphenols and Antioxidant Activity of Negroamaro and Primitivo Wines Obtained with Commercial or Autochthonous Yeast

The content of total polyphenolic compounds in wines obtained with native yeasts was higher than that of commercial wines. The increases ranged from 1.28- to 1.48-fold in Primitivo wines (PA2 and P1A-P3A, respectively) (Table 2) and from 1.1- to 1.45-fold (N2A and N1A, respectively) in Negroamaro wines with an intermediate value of increase in N3A (1.3-fold Table 3).

Significant increase in total antioxidant activity (AA) has been observed with all the selected yeast strains used. In Primitivo wines produced by selected yeast was observed the higher AA value in P1A (1.49-fold increase) and in N3A for Negroamaro wines (1.83-fold increase). In these two wines, we found the flavonols and soluble acids higher than in the other wines. These results suggest a parallelism with the higher content of polyphenol groups and with their synergic activity.

An increasing number of investigations have studied the role of different wine yeast strains and of enological additives of microbial origin to improve the contents of phenolic compounds in red wines [34,35].

Our results are consistent with those already described by Carew and coworkers [16], who demonstrated that a different polyphenols content and enhanced concentration of total and not bleachable pigment of Pinot noir wines was produced by different yeast starter strains. The results obtained here indicated that autochthonous yeast strains are able to considerably modify the polyphenol composition of the produced wines. These findings corroborate the results obtained by Tufariello and co-workers [21], who recently recognized a correlation between the chemical profile of wine and the yeast starter used during vinification, showing that the specific genetic properties of a yeast culture can determine the color and the chemical composition of the obtained wine.

### 3.4. Comparison of Vascular Anti-Inflammatory Response of Polyphenol Extracts from Negramaro and Primitivo Wines Obtained with Commercial or Autochthonous Yeast

Observational and nutritional trials confirm the valuable role of moderate consumption of wine, especially red wine, on the prevention and treatment of chronic non-communicable diseases, such as cardiovascular disease, metabolic syndrome and its components and highlight a positive effect on cardiovascular events and mortality [36].

Although it is known that alcohol favorably modifies the lipid pattern, the reduction of cardiovascular risk seems to be largely related to the effect of non-alcoholic components, mainly resveratrol and other polyphenols, on the vascular wall and on blood cells [36]. In addition to the antioxidant actions, the beneficial effects of red wine polyphenols on vascular function are due to their ability to modulate endothelial–monocyte interactions involved in atherosclerosis [36].

The start of atherosclerosis is composed of the recruitment and adhesion of circulating monocytes to endothelial cells and subsequent trans-endothelial migration into the intima of the vascular wall [37]. This last process involves the concerted expression on the surface of the activated endothelium of adhesion molecules, such as vascular cell adhesion molecule-1 (VCAM-1) which recognizes and binds to the counter-receptor VLA4 on monocytes, thus highlighting a crucial role of VCAM-1 in the recruitment of monocytes in the atherosclerotic process. The subsequent monocyte trans-endothelial migration is mediated by the proteolytic degradation of extracellular matrix by matrix metalloproteinases (MMP)-9 and -2. Recent studies have documented the ability of polyphenol extracts from Negroamaro and Primitivo wine and grape to abrogate the adhesion of human monocytes to inflamed endothelial cells by inhibiting endothelial adhesion molecules expression, as well as MMP activity and expression involved in vascular remodeling and monocyte invasion [9,25].

In this study, we have analyzed the vascular anti-inflammatory properties of polyphenolic extracts obtained from Negroamaro or Primitivo wine produced with autochthonous or industrial yeast strains. To this aim we achieved blends of wines of Negroamaro, NC (N1C, N2C, N3C blend), or Primitivo, PC (P1C, P2C, P3C blend), produced with commercial yeast or obtained by autochthonous yeast from Negroamaro, NA (N1A, N2A, N3A blend), or Primitivo, PA (P1A, P2A, P3A blend), respectively. In Table 4, the total phenol and antioxidant capacity of wine polyphenolic extracts obtained from different blends of Negroamaro (NC, NA) or Primitivo wine (PC, PA) are reported. The data indicated that both wines obtained with autochthonous yeast exhibited higher antioxidant capability and polyphenolic content, compared to those obtained with commercial yeasts.

**Figure 2:**
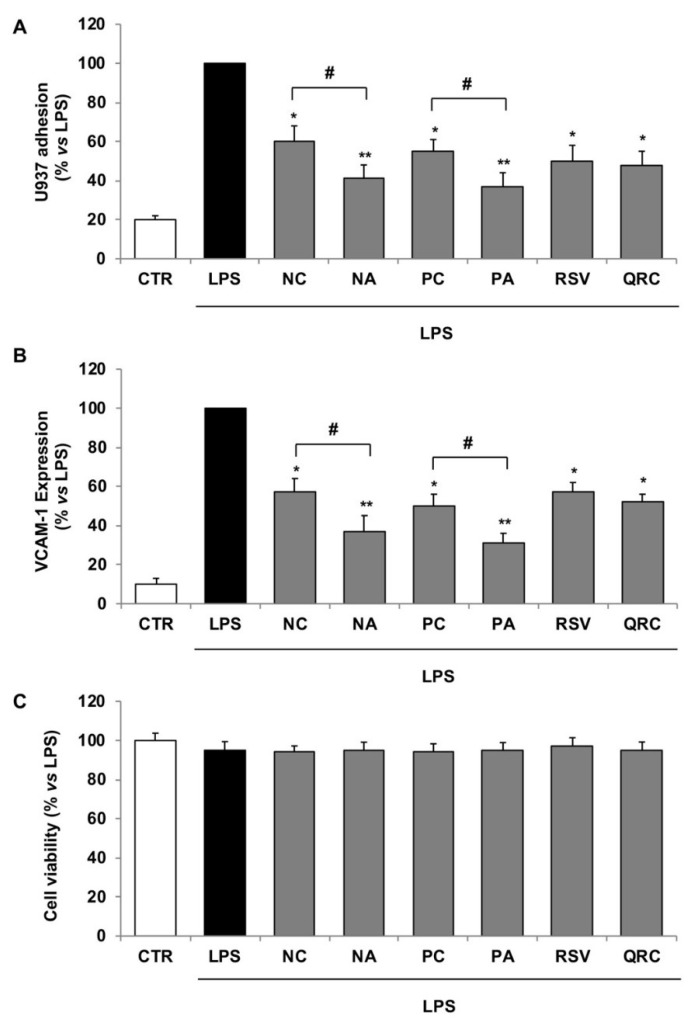
Effects of wine polyphenolic extracts or pure polyphenols on endothelial–monocyte adhesion and VCAM-1 expression. HMEC-1 were incubated with blend polyphenolic extracts (NC, PC, NA, PA) (10 µL), pure polyphenols (resveratrol, RSV; quercetin, QRC) (10 µmol/L), or vehicle (control, CTR) for 1 h and then stimulated with LPS 0.5 μg/mL for 16 h. (**A**): HMEC-1 were co-cultured with calcein AM-labeled U937 monocytes. Fluorescence plate reader measured the number of adherent U937 cells. The values of monocyte adhesion are represented in comparison to monocyte adhesion to LPS-stimulated HMEC-1, normalized at 100%. (**B**): Cell surface expression of VCAM-1 was analyzed by cell surface enzyme immune assay (EIA). (**C**): Endothelial cell viability by MTT assay. Each experiment was performed in triplicate. Data are expressed as the percentage of LPS (mean ± S.D.). * *p* < 0.05; ** *p* < 0.01 vs. LPS alone; # *p* < 0.05 NA vs. NC or PA vs. PC.

To compare the vasculo-protective action of different wine blends, we analyzed the effect of polyphenolic extracts obtained from the same amount of wine blend on endothelial–monocyte adhesion, a crucial step in the inflammatory and atherosclerotic process. For this purpose, human endothelial cells were incubated with wine polyphenolic extracts from the Negroamaro or Primitivo blends, containing polyphenol concentrations effective to reduce endothelial–monocyte adhesion [9], before stimulation with the bacterial endotoxin lipopolysaccharide (LPS) a known pro-inflammatory and pro-atherosclerotic trigger. As shown in Figure 2a, monocytes strongly adhered to LPS-stimulated endothelial cells. The one-hour pre-exposure of HMEC-1 with polyphenol extracts (NC, NA, PC, PA) significantly decreased LPS-induced monocyte adhesion. NA and PA showed greater inhibitory effects on endothelial–monocyte adhesion than NC and PC, respectively.

Since the adhesion of monocytes to the endothelium is mediated by the increased expression of endothelial adhesion molecules, we investigated the effect of polyphenol extracts of NA, PA, NC, PC wine blend on the LPS-induced expression of VCAM-1 by cell-surface EIA. The endothelial adhesion molecule VCAM-1, expressed at low levels in unstimulated HMEC-1, was strongly boosted after LPS challenge and significantly lowered by all blend polyphenol extracts (Figure 2b). Similar to the reduction of endothelial–monocyte adhesion, the inhibition potency of VCAM-1 expression was higher for wine blend obtained with autochthonous yeasts (NA and PA) than that produced with commercial yeasts (NC and PC). These results can be explained by polyphenol amount as well as the peculiar content of stilbenes, phenolic acids, and flavonols in wine blends obtained with different yeast fermentations. To validate the vascular effects of wine blend we include a positive control in cell assays, represented by the pure polyphenols, resveratrol and quercetin, which are two well-recognized anti-inflammatory agents [8,9]. We show that resveratrol (RSV) or quercetin (QRC) at 10 μmol/L significantly inhibit endothelial–monocyte adhesion (Figure 2a) and endothelial VCAM-1 expression (Figure 2b), confirming that the protective action of the wine blend on the vascular endothelium is due to the anti-inflammatory action of the constituent polyphenols.

It is noteworthy that the anti-inflammatory property of wine blend polyphenols or pure polyphenols did not affect the endothelial cell viability as determined by MTT assays (Figure 2c), suggesting that the inhibition effects occurred by modulating the LPS-induced inflammatory response in stimulated endothelial cells in absence of any toxicity.

## 4. Conclusions

To make wine successful as a functional beverage, further studies are required in consumer perception of the healthiness of wine, the relationship between taste and health-enhancing properties, and the viticultural and vinification practices that influence polyphenol concentration and composition. The present findings suggest that the exploitation of autochthonous selected yeast strains can increase the concentration of the phenolic compounds and the antioxidant and inflammatory activity in the produced wine. Moreover, this study extends our previous findings about the ability of polyphenolic extract of Negroamaro and Primitivo wines to decrease monocyte adhesion and to stimulate endothelial cells and the expression of endothelial adhesion molecules [9].

This autochthonous fermentation starter can be defined as an “antioxidant positive strain” [38,39]. Our findings stimulate further work on this topic and suggest the use of this positive feature in the selection of *S. cerevisiae* for winemaking.

## Figures and Tables

**Table 1:** Main physical and chemical parameters characterizing Primitivo (P) and Negroamaro (N) wines respectively produced by using the autochthonous yeast starters ITEM 14093 and ITEM 14077. The values are the means of two vinification at industrial scale. TA, Total Acidity; VA, Volatile Acidity. Alcohol concentration are expressed as g/100mL; all other prameters are expressed as g/L.

Wine	Alcohol	Sugars	AT	AV	Malic	Lactic	Tartaric	Citric	Glycerol	Methanol
P1A	13.39 ± 0.003	2.06 ± 0.071	5.86 ± 0.026	0.39 ± 0.004	2.26 ± 0.027	0.26 ± 0.012	2.72 ± 0.048	0.23 ± 0.001	9.77 ± 0.02	0.06 ± 0.002
P2A	15.87 ± 0.011	2.47 ± 0.003	5.53 ± 0.044	0.36 ± 0.010	2.89 ± 0.029	0.19 ± 0.032	2.41 ± 0.058	0.22 ± 0.014	8.89 ± 0.034	0.08 ± 0.006
P3A	14.88 ± 0.104	2.37 ± 0.547	5.93 ± 0.117	0.43 ± 0.041	2.42 ± 0.363	0.29 ± 0.015	3.95 ± 0.042	0.28 ± 0.034	10.01 ± 0.171	0.05 ± 0.008
N1A	13.10 ± 0.009	2.36 ± 0.119	5.81 ± 0.020	0.40 ± 0.004	2.20 ± 0.019	0.27 ± 0.022	2.01 ± 0.009	0.24 ± 0.006	8.56 ± 0.045	0.05 ± 0.005
N2A	13.72 ± 0.018	1.42 ± 0.151	5.22 ± 0.007	0.42 ± 0.080	2.50 ± 0.003	0.32 ± 0.024	2.22 ± 0.014	0.12 ± 0.009	8.67 ± 0.075	0.03 ± 0.001
N3A	13.38 ± 0.034	0	5.29 ± .0.910	0.41 ± 0.040	2.10 ± 0.023	0.17 ± 0.014	2.48 ± 0.015	0.31 ± 0.012	8.41 ± 0.042	0.02 ± 0.002

**Table 2:** Comparison of different classes of polyphenols content in Primitivo wine estracts (PC) fermented with commercial yeast and Primitivo wine extracts obtained by utilizing selected autochthonous yeast strain (PA). Comparison of Total Phenolics content (TP) and Antioxidant Activity (AA) in Primitivo wine extracts (PC and PA). Values reported in the table are the mean ± SD of three experiments.

Groups	Compounds	P1C	P1A	P2C	P2A	P3C	P3A
		mg/L					
*STILBENES*	trans-Resveratrol	3.7 ± 0.04	18.4 ± 0.31 *	3.0 ± 0.07	23.0 ± 0.31 *	0.9 ± 0.01	7.2 ± 0.06 *
	trans-Piceid	9.4 ± 0.01	41.8 ± 0.09 *	9.2 ± 0.09	44.8 ± 0.27 *	2.8 ± 0.095	11.2 ± 0.09 *
	total	*13.1*	*60.2*	*12.2*	*67.8*	*3.7*	*18.4*
*PHENOLIC ACIDS*	Caftaric acid	522.3 ± 5.9	1044.1 ± 3.1 *	558.9 ± 4.87	1050 ± 6.9 *	354.0 ± 3.72	637.0 ± 3.1 *
	Caffeic acid	30.0 ± 1.8	180.7 ± 1.0 *	69.0 ± 0.7	180.5 ± 4.8 *	69.7 ± 2.11	88.3 ± 2.11 *
	p-Coumaric acid	6.4 ± 0.11	6.6 ± 0.7	4.32 ± 0.48	3.8 ± 0.02	3.8 ± 0.48	5,7 ± 2,8 *
	total	*558.7*	*1226.4*	*632.22*	*1233.8*	*427.57*	*731.0*
*FLAVONOLS*	Myricetin	3.3 ± 1.0	30.4 ± 1.4 *	4.6 ± 0.45	25.2 ± 3.1 *	7.0 ± 0.11	8.5 ± 0.95 *
	Quercetin	8.4 ± 0.7	46.9 ± 8.6 *	9.9 ± 0.68	8.6 ± 1.0	7.6 ± 0.05	28.6 ± 0.8 *
	Kaempferol	4.5 ± 0.1	11.5 ± 0.98 *	5.4 ± 0.11	8.6 ± 0.7 *	5.7 ± 0.02	7.9 ± 0.6 *
	total	*16.2*	*88.8*	*19.9*	*42.4*	*20.3*	*45.0*
*FLAVANOLS*	Catechin	8.8 + 0.2	8.9 ± 0.1	8.0 ± 0.1	8.5 ± 0.2	8.30 ± 0.5	8.9 ± 0.2
	Epicatechin	9.4 ± 0.5	10.2 ± 0.5	8.07 ± 0.4	9.0 ± 0.4	9.5 + 0.51	9.2 + 1.1
	total	*18.2*	*19.1*	*16.07*	*17.5*	*17.8*	*18.1*
** TP	mg GAEs/L	928.8 ± 9.0	1366.6 ± 8.0 *	1221.9 ± 7.6	1569.3 ± 7.6 *	925.7 ± 6.8	1376.2 ± 8.0 *
*§ AA (TEAC)*	mmol TE/100ml	64.6 ± 1.1	96.4 ± 1.5 *	77.3 ± 0.6	95.9 ± 1.7 *	76.6 ± 2.1	96.03 ± 1.8 *

* Statistically different values, Student’s *t*-test *p*-value < 0.05; ** Total Phenolics are expressed as mg of Gallic Acid equivalents per Liter. § Antioxidant activity is expressed as mmoles of Trolox equivalents per 100 mL.

**Table 3:** Comparison of different classes of polyphenols content in Negroamaro wine extracts fermented with commercial yeast (NC) and Negroamaro wine extracts obtained by utilizing selected autochthonous yeast strain (NA). Comparison of Total Phenols content (TP) and Antioxidant Activity (AA) in Negroamaro wine extracts (NC and NA). Results reported in table are expressed as the mean ± SD of three experiments.

Groups	Compounds	N1C	N1A	N2C	N2A	N3C	N3A
		mg/L					
*STILBENES*	trans-Resveratrol	4.8 ± 0.04	4.7 ± 0.05	3.2 ± 0.05	10.7 ± 0.7 *	1.6 ± 0.04	7.8 ± 0.1 *
	trans-Piceid	14.03 ± 0.1	47.2 ± 1.0 *	7.7 ± 0.1	28.9 ± 0.9 *	5.5 ± 0.03	15.5 ± 0.08 *
	total	*18.83*	*51.9*	*10.9*	*39.6*	*7.1*	*23.3*
*PHENOLIC ACIDS*	Caftaric acid	574.5 ± 5.0	1373 ± 7.0 *	484.3 ± 5.0	1633.9 ± 6.9 *	293.5 ± 3.7	1267.2 ± 3.1 *
	Caffeic acid	96.9 ± 1.8	304.8 ± 1 *	89.5 ± 0.7	334.8 ± 4.8 *	53.5 ± 2.0	644.5 ± 2.1 *
	p-Coumaric acid	6.4 ± 0.1	6.0 ± 0.4	4.3 ± 0.5	8.0 ± 0.02 *	3.9 ± 0.98	5.7 ± 0,85
	total	*677.8*	*1683.8*	*578.1*	*1976.7*	*350.9*	*1917.4*
*FLAVONOLS*	Myricetin	8 ± 0.1	7.8 ± 1.4	9.4 ± 0.45	14.1 ± 0.3 *	0.37 ± 0.02	5.4 ± 0.9 *
	Quercetin	24.4 ± 0.7	23.3 ± 0.6	14.3 ± 0.7	65.9 ± 1 *	1.3 ± 0.05	10 ± 0.8 *
	Kaempferol	17.4 ± 0.1	26.8 ± 0.9 *	3.3 ± 0.1	26.1 ± 0.7 *	0.61 ± 0.02	1.70 ± 0.6 *
	total	*49.8*	*57.9*	*30.3*	*106.1*	*2.28*	*17.1*
*FLAVANOLS*	Catechin	8.0 ± 2.2	8.0 ± 0.1	8.2 ± 0.1	7.5 ± 0.2	7.30 ± 0.2	*6.98 ± 0.5*
	Epicatechin	10.23 ± 2.12	12.2 ± 2.0	12.7 ± 2.2	15.0 ± 2.4	10.05 ± 2.0	11.21 ± 1.1
	total	*18.23*	*20.2*	*20.9*	*22.5*	*17.35*	*18.19*
** TP	mg GAEs/L	860.2 ± 9.0	1255.2 ± 8.0 *	1118.5 ± 7.6	1245.5 ± 7.6 *	971.2 ± 6.8	1277.3 ± 8.0 *
*§ AA (TEAC)*	mmol TE/100ml	62.9 ± 1.1	83 ± 1.5 *	56.3 ± 0.6	83 ± 1.7 *	50.3 ± 2.10	92.2 ± 1.8 *

* Statistically different values, Student’s *t*-test *p*-value < 0.05; ** Total Phenolics are expressed as mg of Gallic Acid equivalents per Liter. § Antioxidant activity is expressed as mmoles of Trolox equivalents per 100 mL.

**Table 4:** Comparison of Total Phenolics content (TP) and Antioxidant Activity (AA) in Negramaro (NC and NA) and Primitivo wine extracts (PC and PA). Values reported in the table are the mean SD of three experiments.

	NC	NA	PC	PA
** TP (mg GAEs/L)	983.3 ± 9.5	1296.5 ± 10.3 *	1025.5 ± 12.3	1102.1 ± 8.4
§ AA (mmol TE/L)	565.3 ± 5.4	905.2 ± 7.8 *	728.4 ± 6.5	961.3 ± 7.2 *

* Statistically different values, Student’s *t*-test *p*-value < 0.05; ** Tatal Pjenolics are expressed as mg of Gallic Acid equivalents per Liter. § Antioxidant activity is expressed as mmoles of Trolox equivalents per Liter.
